# Exopolysaccharide is the potential effector of *Lactobacillus fermentum* PS150, a hypnotic psychobiotic strain

**DOI:** 10.3389/fmicb.2023.1209067

**Published:** 2023-07-03

**Authors:** Chin-Lin Huang, Hsu-Feng Chu, Chien-Chen Wu, Fu-Sheng Deng, Pei-Jun Wen, Shao-Ping Chien, Chi-Hsein Chao, Ying-Tsong Chen, Mei-Kuang Lu, Ying-Chieh Tsai

**Affiliations:** ^1^Biomedical Industry Ph.D. Program, National Yang Ming Chiao Tung University, Taipei, Taiwan; ^2^Bened Biomedical Co., Ltd., Taipei, Taiwan; ^3^Institute of Genomics and Bioinformatics, National Chung Hsing University, Taichung, Taiwan; ^4^National Research Institute of Chinese Medicine, Ministry of Health and Welfare, Taipei, Taiwan; ^5^Institute of Molecular and Genomic Medicine, National Health Research Institutes, Zhunan Town, Miaoli County, Taiwan; ^6^Institute of Biochemistry and Molecular Biology, National Yang Ming Chiao Tung University, Taipei, Taiwan

**Keywords:** exopolysaccharide, EPS, heat-killed, Lactobacillus fermentum, PS150, hypnotic, postbiotic

## Abstract

Psychobiotics are a class of probiotics that confer beneficial effects on the mental health of the host. We have previously reported hypnotic effects of a psychobiotic strain, *Lactobacillus fermentum* PS150 (PS150), which significantly shortens sleep latency in experimental mice, and effectively ameliorate sleep disturbances caused by either caffeine consumption or a novel environment. In the present study, we discovered a *L. fermentum* strain, GR1009, isolated from the same source of PS150, and found that GR1009 is phenotypically distinct but genetically similar to PS150. Compared with PS150, GR1009 have no significant hypnotic effects in the pentobarbital-induced sleep test in mice. In addition, we found that heat-killed PS150 exhibited hypnotic effects and altered the gut microbiota in a manner similar to live bacteria, suggesting that a heat-stable effector, such as exopolysaccharide (EPS), could be responsible for these effects. Our comparative genomics analysis also revealed distinct genetic characteristics in EPS biosynthesis between GR1009 and PS150. Furthermore, scanning electron microscopy imaging showed a sheet-like EPS structure in PS150, while GR1009 displayed no apparent EPS structure. Using the phenol-sulfate assay, we found that the sugar content value of the crude extract containing EPS (C-EPS) from PS150 was approximately five times higher than that of GR1009, indicating that GR1009 has a lower EPS production activity than PS150. Through the pentobarbital-induced sleep test, we confirmed the hypnotic effects of the C-EPS isolated from PS150, as evidenced by a significant reduction in sleep latency and recovery time following oral administration in mice. In summary, we utilized a comparative approach to delineate differences between PS150 and GR1009 and proposed that EPS may serve as a key factor that mediates the observed hypnotic effect.

## 1. Introduction

Probiotics are live microbes that can have beneficial effects on the host. Owing to their high commercial value, studies assessing various functions of probiotics have considerably advanced over the past decade (Kerry et al., 2018). One of the most important milestones is the theory of “psychobiotics,” which further extends the function of probiotics to behavior and mental health (Dinan et al., 2013). Given the accumulating evidence on the microbiota-gut-brain axis, the definition of psychobiotics has been extended to include prebiotic molecules featuring the ability to promote the growth of gut microbiota (Sarkar et al., 2016). In addition to the discovery of novel applications, numerous probiotic functions were found to correlate with non-viable cells, resulting in a surge of research on postbiotics (de Almada et al., 2016; Lebeer et al., 2018; Teame et al., 2020).

Postbiotics are defined as “preparations of inanimate microorganisms and/or their components that confer a health benefit on the host” (Salminen et al., 2021). A postbiotic may be composed of intracellular proteins, cell wall components, cell surface associated molecules, exopolysaccharides (EPS), secretory molecules, and/or bacterial metabolites. These bioactive molecules are ideal starting points for elucidating underlying mechanisms due to their relatively simple nature (Plaza-Diaz et al., 2019). Several cellular receptors, including Toll-like receptors (Murofushi et al., 2015; Plovier et al., 2017; Salminen et al., 2021), nucleotide-binding oligomerization domain-like receptors (Shida et al., 2009), c-type-lectin receptors (Konstantinov et al., 2008; Tytgat et al., 2016), and G-protein-coupled receptors (Brown et al., 2003; Hong et al., 2005) are reportedly sensitive to these molecules.

Among molecules isolated from microbes, EPS is particularly interesting, owing to its diverse chemical properties, heat stability, and potential roles in host–microbe interactions (Lee et al., 2016). The highly diverse chemical compositions containing various EPS can be employed in industrial applications, such as emulsifiers, food additives, antioxidants, cryoprotectants, and nanoparticle stabilizers (Zannini et al., 2016; Wang et al., 2019; Zhou et al., 2019). One of the most well-studied probiotic genera, *Lactobacillus*, is frequently used to isolate bioactive EPS (Zhou et al., 2019). As a common specie in various fermented foods, *Lactobacillus fermentum* is known for its slimy texture and EPS-producing capability, making it an ideal source for novel EPS discovery. EPS-producing *L. fermentum* strains, such as Lf2 and MTCC 25067, have been extensively investigated for their chemical structure, genetics, rheological properties, and EPS production (Ale et al., 2016; Aryantini et al., 2017; Ikeda et al., 2019; Vitlic et al., 2019; Mengi et al., 2020; Ale et al., 2020a). More importantly, available preliminary evidence links the probiotic function of *L. fermentum* strains to EPS (Ale et al., 2020b).

Previously, we had reported that *L. fermentum* PS150 (PS150) is a psychobiotic that could remodel the host microbiota and ameliorate caffeine-induced sleep disturbances (Lin et al., 2019) or the first-night effect (Lin et al., 2021). In the present study, we identified another *L. fermentum* strain, GR1009, with a distinct colony morphology as compared with PS150 isolated from the same source. Colony morphology has long been a powerful indicator of microbial physiology. Distinct colony morphologies between bacterial strains can be linked to differences in surface molecules, virulence, and biofilm formation (Kansal et al., 1998; Martin-Rodriguez et al., 2021). Although PS150 and GR1009 are genetically similar to each other, GR1009 exhibits an impaired hypnotic effect. Using a genomics analysis, we characterized differences between PS150 and GR1009. Furthermore, EPS was found to be a potential effector of the PS150-mediated hypnotic effect. The present study highlights the mechanism of PS150 and the possible application of the PS150-derived EPS as a hypnotic drug in future.

## 2. Materials and methods

### 2.1. Preparation of bacteria

*Lactobacillus fermentum* (recently re-classified as *Limosilactobacillus fermentum*; Zheng et al., 2020) strains PS150 and GR1009 were both isolated from the same fermented sausage (Liu et al., 2019). Bacterial strains used in the present study were anaerobically cultured in Mann Rogosa Sharp (MRS) broth (Criterion, Hardy Diagnostics, Santa Maria, CA, USA) at 37°C for 18 h. The cells were harvested by centrifugation at 4°C, 10,000 *g* for 10 min. For the animal model, the cells were resuspended in MRS broth containing 12.5% glycerol and adjusted to a final concentration of 10^10^ colony-forming units per milliliter (CFU/ml), and then aliquoted and stored at −80°C. Before oral administration, bacterial stocks were removed from −80°C storage and thawed in water bath at 37°C for 1 h and then centrifuged at 10,000 *g* for 10 min at 4°C. The supernatant was discarded, and the pellet was resuspended in phosphate-buffered saline (PBS). For heat-killed *L. fermentum* PS150 (HK-PS150), the harvested cells were resuspended in PBS and adjusted to a final concentration of 10^10^ CFU/ml, and then heated at 80°C for 30 min in the water bath. The heat-treated samples were also stored at −80°C before use.

### 2.2. Genomic DNA fingerprinting of bacterial strains

Bacterial genomic DNA was prepared by phenol extraction. briefly, the harvested cell pellets were resuspended in genome extraction buffer (200 mM Tris–HCl, 80 mM EDTA (pH 9.0), 2% w/v sodium dodecyl sulfate). The suspensions were then supplied with an equal volume of phenol and 0.1 mm glass beads. The mixtures were lysed with a FastPrep FP120 homogenizer (Q-Biogene, Carlsbad, CA, USA), and genomic DNA was extracted using phenol-chloroform extraction. The quality of DNA extracts was validated using NanoDrop spectrophotometer. Random amplification of polymorphic DNA (RAPD) and enterobacterial repetitive intergenic consensus (ERIC) PCR were performed using Takara Taq polymerase (Takara Bio Inc., Shiga, Japan) with designated primers In accordance with the manufacturer’s instructions (Versalovic et al., 1991). The arbitrary sequence RAPD-B (5′-AACGCGCAAC-3′) was used in RAPD, and the primer pair ERIC1 (5′-ATGTAAGCTCCTGGGGATTCAC-3′) and ERIC2 (5′-AAGTAAGTGACTGGGGTGAGCG-3′) were used in ERIC PCR. RAPD and ERIC PCR products were analyzed by electrophoresis In a 1% agarose gel, followed by SYBR safe staining (Thermo fisher, Waltham, MA, USA).

### 2.3. Next generation sequencing (NGS) library preparation and data analysis

The bacterial culture was spread on MRS agar plates and incubated anaerobically at 37°C for 48 h. After incubation, colonies were scraped from the agar plates for DNA extraction. A DNeasy Blood and Tissue Kit (Qiagen, Hilden, Germany) was used to extract genomic DNA for MinION long-read sequencing. For Illumina sequencing, DNeasy UltraClean microbial kits (Qiagen) were used to prepare genomic DNA. Genomic DNA was extracted according to the manufacturer’s protocol provided by the vendor. The quality of extracted DNA was examined using Qubit4 (Thermo Fisher, Waltham, MA, USA) and a UV spectrophotometer. The shotgun sequencing library for Illumina sequencing was prepared using the Nextera DNA Flex library with DNA CD Indexes (Illumina, CA, USA) following standard protocols. Quality control of the sequencing libraries was performed using an Agilent 2,100 Bioanalyzer (Agilent, CA, USA). Sequencing was performed using an iSeq100 (Illumina). De-multiplexing and trimming were performed using a Basespace (Illumina). For long-read sequencing, a shotgun library for genomic DNA was constructed using the Rapid Barcoding kit SQK-RBK004 (Oxford Nanopore Technologies, Oxford, UK). The sequencing library was sequenced, base- called, and debarcoded on a MinION Mk1C (Oxford Nanopore Technologies). *De novo* genome assembly was performed using Unicucler v0.4.8 (Wick et al., 2017). The completeness of the resulting assembly was validated using Bandage (Wick et al., 2015) and further examined by mapping Illumina short reads using the CLC Genomic Workbench (Qiagen). The genomes were annotated using the NCBI Prokaryotic Genome Annotation Pipeline. Carbohydrate gene clusters were analyzed using the dbCAN2 meta server and MAUVE (Darling et al., 2004; Yin et al., 2012; Zhang et al., 2018). The CLC Genomics Workbench (Qiagen) was used for single nucleotide polymorphism (SNP) analysis. To identify inverted repeat sequences, the flanking sequence of the IS256 transposase was analyzed using the palindrome function of the EMBOSS server (Rice et al., 2000).

### 2.4. PCR amplification and sequencing of *eps1* cluster region

The PCR primer set Eps1F (5′-ATCCCACCCACATGACGTTC-3′) and Eps1R (5′-AGTTTATCCGCACGAGGAGT-3′) were designed according to specific DNA sequences (located in 113,863–113,882 and 128,276 – 128,295) respectively in the chromosome of PS150. The estimated amplicon of Eps1F/Eps1R was 14,433 bp in PS150 and 2,303 bp in GR1009. Amplification of *eps1* was carried out using a long PCR enzyme mix (Thermo Fisher, Waltham, MA, USA) following the manufacturer’s three-step cycling protocol. First, the molecular size of the PCR products was analyzed by electrophoresis in 1% agarose gel, followed by SYBR safe staining (Thermo Fisher, Waltham, MA, USA). The DNA sequence of the PCR products was then bidirectionally confirmed by Sanger sequencing using the Eps1F and Eps1R primer set.

### 2.5. Animals

Male C57BL/6J mice (6 weeks old) were purchased from the National Laboratory Animal Center (Taipei, Taiwan). The mice were housed in the Laboratory Animal Center of the National Yang Ming Chiao Tung University. The room was maintained at a constant temperature (22 ± 1°C) and humidity (55–65%) with a 12 h light/dark cycle. The mice were fed *ad libitum* with a standard chow diet and sterilized water. All experiments were conducted in accordance with relevant guidelines and regulations, and were approved by the Institutional Animal Care and Use Committee of National Yang Ming Chiao Tung University (IACUC No. 1080702).

### 2.6. Pentobarbital-induced sleep test

For the comparative assessment of PS150, GR1009 and HK-PS150, the bacterial stocks were thawed and recovered at room temperature before use. The time- and dose-dependent properties of PS150 have been confirmed, the mice used in this experiment were intragastrically administered 0.2 ml PBS or bacterial suspensions (10^9^ CFU/day) for 13 days (Lin et al., 2019). On day 14, the mice were administered PBS, 10 mg/kg diphenhydramine (DIPH, Sigma, Saint Louis, MO, USA), or bacterial suspensions 30 min before the intraperitoneal injection of pentobarbital sodium (50 mg/kg, Sigma). Following the injection, the mice were tested for righting reflex by flipping upside down. The time between the pentobarbital injection and the loss of righting reflex was defined as the sleep latency. The time required to restore the righting reflex was defined as the sleep duration, whereas the time spent between righting action and voluntary movement was defined as the recovery time. For the functional validation of (C-EPS), the mice were orally administered PBS, MRS broth, or C-EPS (0.4 mg/day) for 13 days. On day 14, the mice were treated as described above for evaluating sleep conditions.

### 2.7. Fecal sample collection and DNA extraction

All mice were placed individually in clean cages until defecation. Immediately after the mice defecated, the stool samples were collected into 1.5 ml microtubes containing 0.3 ml of RNAlater solution (Invitrogen) and then frozen at −80°C. For DNA extractions, samples were homogenized and washed twice with PBS and then lysed with 0.2 mm glass beads using a FastPrep FP120 homogenizer (Q-Biogene, Carlsbad, CA, USA). Following centrifugation at 12,000 *g* for 5 min at 4°C, then the supernatant (0.4 ml) was collected. The bacterial genomic DNA was extracted using phenol–chloroform extraction, and subsequently, the concentration and quality of the DNA were determined using the NanoDrop ND-1000 spectrophotometer (Thermo Fisher Scientific, Waltham, MA, USA).

### 2.8. Microbiota analysis

Next generation sequencing and data processing were performed by BioTools Co. Ltd. (Taipei, Taiwan). Briefly, the 16S rRNA V3–V4 region was amplified via PCR using a region-specific primer set (341F: 5′-CCTACGGGNGGCWGCAG-3′, 806R: 5′- GACTACHVGGGTAT CTAATCC-3′) according to the 16S Metagenomic Sequencing Library Preparation procedure (Illumina). The quality of the indexed PCR product was evaluated using the Qubit 4.0 Fluorometer (Thermo Fisher Scientific, Waltham, MA, USA) and Qsep100™ system (Bioptic). An equal amount of the indexed PCR product was combined to create the sequencing library, which was ultimately sequenced on an Illumina MiSeq platform to generate paired 300-bp reads. After demultiplexing each sample, the primer and adapter sequences were removed from the paired-end reads using the QIIME2 cutadapt plugin. To construct the Amplicon Sequence Variants (ASVs), a denoising pipeline was executed using the QIIME2 DADA2 plugin (v2020.11). The community composition was differentially analyzed using the linear discriminant analysis effect size (LEfSE) method (Segata et al., 2011), which employs a non-parametric Kruskal-Wallis test and Wilcoxon rank-sum test algorithm to identify bacterial taxa whose relative abundance significantly differs between control and experimental groups. Following identification of these taxa, LEfSe uses Linear discriminant analysis (LDA) to assess the effect size of each differentially abundant taxon. In the present study, taxa with LDA score (log_10_) > 2 was considered significant.

### 2.9. Scanning electron microscopy

The bacterial suspensions of PS150 and GR1009 were dispensed and grown on glass slides placed in a 24-well plate. After centrifugation at 2,000 *g* for 5 min, the samples were primarily fixed by 100 mM PBS (pH 7.2) containing 2.5% glutaraldehyde at room temperature for 1 h. The samples were rinsed three times with 5 min incubations in PBS and subsequently fixed by PBS containing 4% paraformaldehyde and 2.5% glutaraldehyde at room temperature for 30 min. After rinsed three times with 5 min incubations in the PBS, the samples were incrementally dehydrated in a series of washes in 30 and 50% ethanol for 10 min each at room temperature, 75% ethanol overnight at 4°C, 85 and 95% ethanol for 10 min each at room temperature, and 100% ethanol three times for 20 min each at room temperature. Finally, the samples were dried by critical-point drying in liquid CO_2_ and sputter-coated with gold and observed by the scanning electron microscopy (JEOL JSM-7600F).

### 2.10. Purification of exopolysaccharide-containing crude extract

Bacterial culture of PS150 was prepared as previously described (Liu et al., 2019). The overnight culture was heated at 80°C for 1 h, and the bacterial cells were removed by centrifugation at 7,000 *g* for 30 min. The polysaccharide component was then precipitated using 3× volume of anhydrous ethanol at 4°C for 24 h. The precipitants were then collected at 7,000 *g* for 30 min and washed with 70% ethanol. The residual ethanol was removed by evaporation, and the EPS-containing extract was dissolved in ddH_2_O and stored at −80°C until use. The recovery rate of saccharide content and protein contamination during the purification process were monitored using phenol sulfuric acid (Masuko et al., 2005) and the Bradford method, respectively.

### 2.11. Data availability

The assembled genomes of PS150 (GR1008) and GR1009 were uploaded to the NCBI Bioproject (PRJNA702613).

### 2.12. Statistical analysis

Results were described as mean ± standard error of the mean (SEM). Graphs were generated and statistical analysis performed using the software GraphPad Prism 9.0. All data were analyzed by one-way ANOVA using the Tukey *post hoc* test or unpaired t-test.

## 3. Results

### 3.1. Characterization of GR1009, a phenotypic variant strain of *Lactobacillus fermentum* PS150

**Figure 1:**
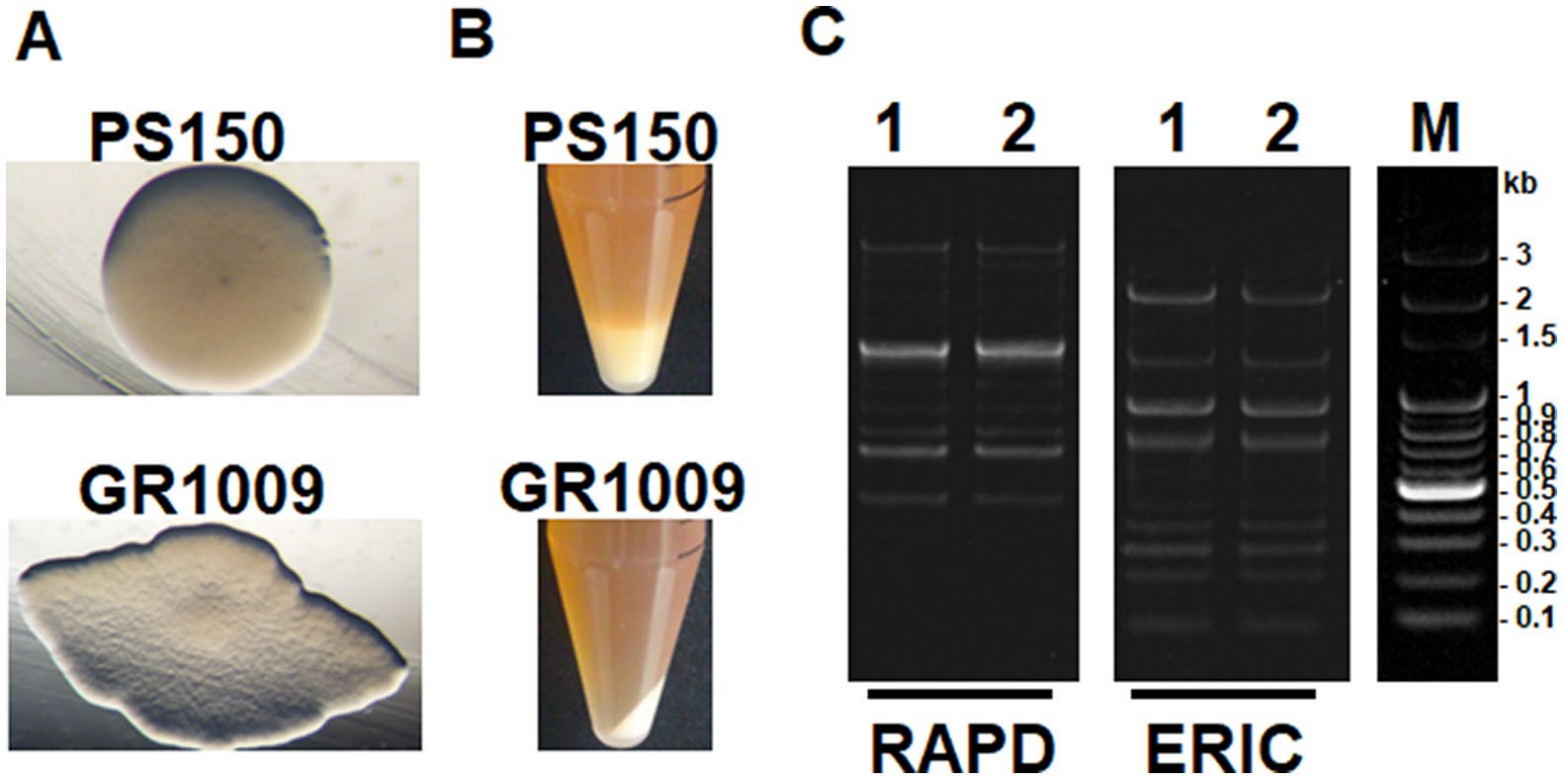
Strain-specific morphotypes and genomic DNA fingerprinting of *L. fermentum* PS150 and GR1009. **(A)** Colony morphologies of PS150 and GR1009. Both strains were spread on MRS agar plates and incubated anaerobically for 48 h. **(B)** Liquid culture of the two strains after centrifugation at 3,000 × *g* for 10 min. **(C)** Random amplified polymorphic DNA (RAPD) and enterobacterial repetitive intergenic consensus (ERIC) PCR fingerprinting profiles of PS150 and GR1009. 1, PS150; 2, GR1009; M, DNA molecular size marker.

PS150 exhibited a white, opaque, and circular colony when cultured anaerobically on a MRS agar plate (Figure 1A). From the same source as PS150, we isolated another *L. fermentum* strain that exhibited a flat, transparent colony with a rough surface and undulated margin, named GR1009. In contrast to the apparent “ropy” phenotype of PS150, the colony of GR1009 was not slimy. Furthermore, after overnight anaerobic incubation in MRS broth, the viscosity of the liquid culture was assessed by centrifugation. PS150 cells formed a puffy layer after centrifugation whereas GR1009 cells could be sedimented into a solid pellet (Figure 1B). Despite apparent differences in colony morphology between PS150 and GR1009, it is intriguing that the DNA fingerprinting profiles thereof were similar (Figure 1C), suggesting that both strains share a common origin.

### 3.2. GR1009 exhibits reduced hypnotic efficacy in the pentobarbital-induced sleep mouse model

**Figure 2:**
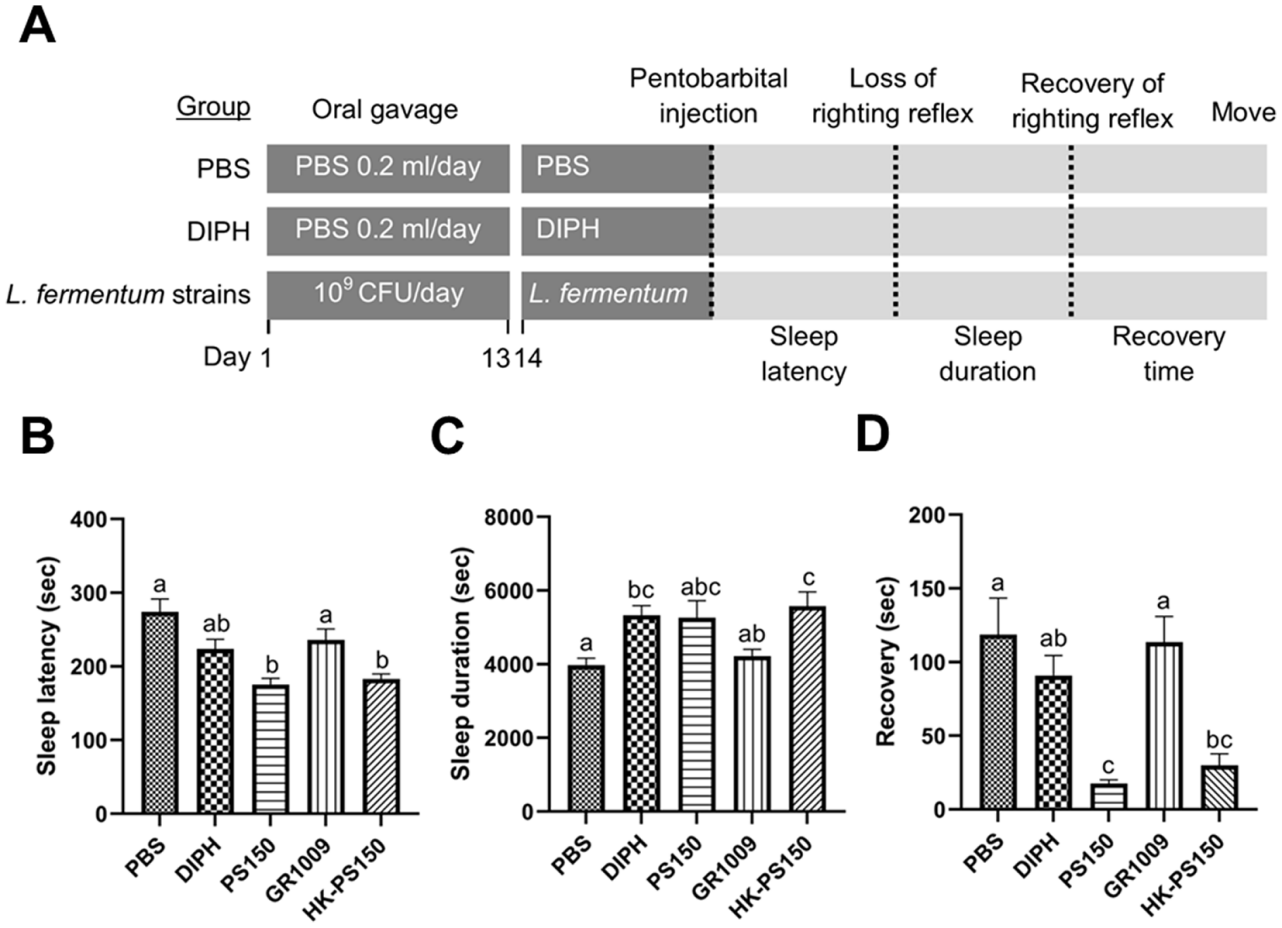
The hypnotic properties of *L. fermentum* PS150, GR1009, and HK-PS150 strains. **(A)** Schematic view of the experimental design. PS150, GR1009, HK-PS150, and PBS were administered to the mice by oral gavage for 14 days. On day 14, pentobarbital was injected to the mice to evaluate sleep parameters. Time required for losing righting reflex after pentobarbital injection, recovering righting reflex, and restoring mobility are defined as sleep latency, sleep duration, and recovery time, respectively. **(B–D)** Effects of PS150, GR1009, or HK-PS150 (*n* = 10 in each group) interventions on the sleep latency, duration, and recovery. Different letters (a, b, or c) above the columns indicate significant differences (*p* < 0.05) between the five groups. Any two treatments are assigned by the same letter at the top of the graphs, it indicates that there is no statistically significant difference. The comparisons were performed using one-way ANOVA with Tukey’s *post hoc* test (*p* < 0.05). The data are expressed as mean ± SEM.

According to our laboratory’s previous study, we have demonstrated the potential sleep-improving effects of PS150 in a pentobarbital-induced sleep mouse model (Lin et al., 2019). In this study, we aimed to investigate whether GR1009 possess the hypnotic efficacy; in addition, we were also interested in the possible effect of PS150 as a postbiotic. We used an 80°C water bath to kill PS150 cells. The PS150, HK-PS150, and GR1009 were then examined in a pentobarbital-induced sleep mouse model to characterize their hypnotic functions. Followed the experiment design, the sleep latency, sleep duration, and recovery time were measured to evaluate the sleep initiation, persistence, and awakening, respectively (Figure 2A). Compared with the PBS group, intervention with PS150 (175.50 ± 26.68 s) and HK-PS150 (183.20 ± 20.79 s) exhibited a significant effect on reducing sleep latency, the reducing effect were more than GR1009 (236.10 ± 46.26 s) and the control drug, DIPH [223.90 ± 40.87 s; Figure 2B, *F*(4, 45) = 10.18, df = 4, *p* < 0.0001]. On sleep duration, both PS150 (5262.00 ± 1465.04 s) and HK-PS150 (5577.10 ± 1228.98 s) groups exhibited a trend in elongating sleep duration, only GR1009 group (4224.33 ± 541.57 s) has no obvious difference compared to the PBS group [3975.70 ± 588.15 s; Figure 2C, *F*(4, 44) = 5.072, df = 4, *p* = 0.0019]. Notably, both PS150 and HK-PS150 exhibited significantly distinct effects on recovery time compared to DIPH and GR1009 [Figure 2D, *F*(4, 43) = 8.587, df = 4, *p* < 0.0001]. Administration of PS150 (17.63 ± 6.95 s) and HK-PS150 (29.90 ± 24.79 s) demonstrated a significant reduction on recovery time, while neither DIPH (90.90 ± 42.99 s) nor GR1009 (113.60 ± 54.56 s) demonstrated this effect. These results suggest that HK-PS150 cells are postbiotics that possess heat-stable bioactive effectors. Furthermore, despite sharing a common origin between PS150 and GR1009, the hypnotic effect of GR1009 was found to be impaired compared to that of PS150. As GR1009 exhibited ineffectiveness of hypnotic activity, we hypothesized that the expression of the bioactive effectors is reduced or absent in GR1009 cells.

### 3.3. Effects of PS150 and HK-PS150 on fecal microbiota composition

**Figure 3:**
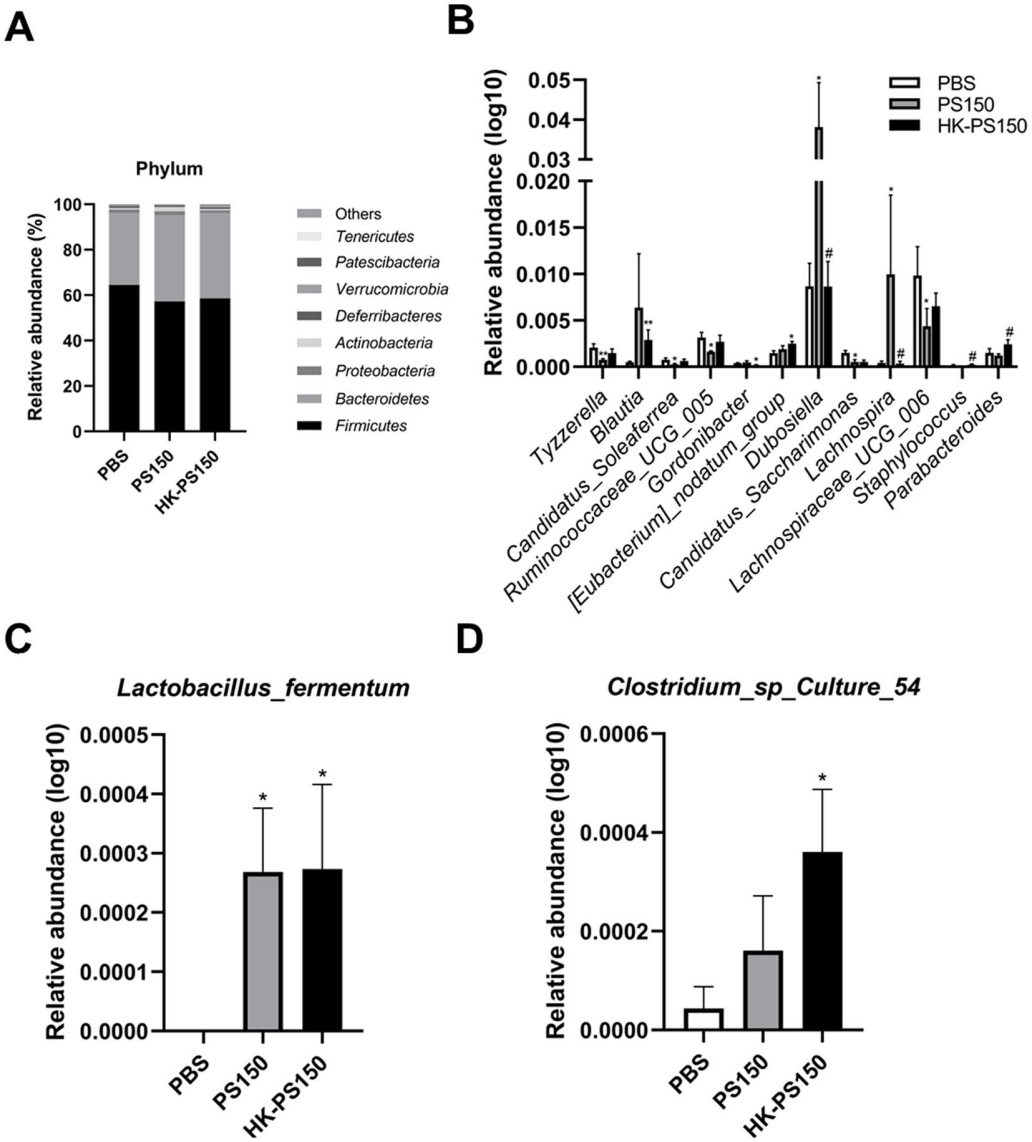
Investigating the effect of PS150 and HK-PS150 supplementation on the microbiota composition. Histogram of relative abundance at the levels of **(A)** phylum, **(B)** genus, **(C)** *Lactobacillus fermentum*, and **(D)** *Clostridium* sp. *Culture* 54.

Next, we investigated the composition of the gut microbiota among the PBS, PS150 and HK-PS150 groups. At the phylum level, *Firmicutes* and *Bacteroidetes* were the most predominant bacterial phyla, the ratio of *Firmicutes/Bacteroidetes* (F/B) have no significant difference between the three experimental groups. Compared to the PBS group, *Actinobacteria* level was increased in PS150 (Fisher’s exact test, *p* = 0.0414) but not in HK-PS150 group. Furthermore, *Bacteroidetes* and *Tenericutes* levels were also slightly increased in both PS150 and HK-PS150 group. In contrast, the relative abundance of *Patescibacteria* significantly decreased in PS150 (Fisher’s exact test, *p* = 0.0127) or HK-PS150 (Fisher’s exact test, *p* = 0.0127) administration (Figure 3A).

At the genus level, we obtained 12 differentially abundant taxa after PS150 or HK-PS150 oral administration. As shown in Figure 3B, the abundances of *Blautia*, *Dubosiella*, and *Lachnospira* were increased in PS150 group, while *Candidatus_Soleaferrea*, *Candidatus_Saccharimonas*, *Lachnospiraceae_UCG_006*, *Ruminococcaceae_UCG_005*, and *Tyzzerella* were decreased compared with the PBS group. Compared with the HK-PS150 group and the PBS group, the relative abundances of *Blautia*, *[Eubacterium]_nodatum_group*, and *Parabacteroides* were increased in the HK-PS150 group while that of *Gordonibacter* was decreased. We then analyzed the gut microbiota composition at the species level in the three experimental groups, *Lactobacillus*_*fermentum* and *Clostridium* sp. *Culture*_54 levels were significantly increased after PS150 or HK-PS150 administration (Figures 3D,E). LEfSe analysis was further applied and identified different microbiome signatures between groups, revealing the gut microbiota composition was altered in both PS150 and HK-PS150 treated mice (Supplementary Figure S1).

### 3.4. Comparative genomic analysis of PS150 and GR1009

We utilized a comparative approach to delineate genomic differences between PS150 and GR1009. Whole-genome sequences of both PS150 and GR1009 were determined using a combination of Nanopore and Illumina sequencing. The results revealed that the genome size of GR1009 was slightly smaller than that of PS150, with approximately the same GC content (Supplementary Table S1). No plasmids were detected in either strain. Single nucleotide polymorphism (SNP) analysis was performed by mapping Illumina sequencing reads of both strains to their assembled genomes. We discovered a total of 11 SNPs, wherein five SNPs were non-synonymous polymorphisms (Table 1). In addition, we identified five insertion–deletion mutations (indels) using whole-genome BLAST, wherein three indels were insertion sequence (IS) elements (Table 2).

**Table 1:** Identification of single nucleotide polymorphisms in *L. fermentum* GR1009.

Location in PS150	Alleles	a.a change	Locus tag of PS150	Putative function
400,514	A → G	E366G	JYQ65_01920	Acetyl-CoA carboxylase biotin carboxylase subunit
601,764	T → C	I97T	JYQ65_02890	Insulinase family protein
667,456	A → C	-	JYQ65_03295	Methyltransferase domain-containing protein
829,195	C → T	S180L	JYQ65_04200	Hypothetical protein
840,587	C → T	P99S	JYQ65_04285	IS3 family transposase
1,152,710	A → C	-	JYQ65_05850	IS256 family transposase
1,158,682	G → A	-	JYQ65_05885	ISL3 family transposase
1,438,686	T → C	-	JYQ65_07305	IS256 family transposase
1,741,305	T → C	-	JYQ65_08910	IS3 family transposase
1,766,646	C → T	-	JYQ65_09045	SAM-dependent DNA methyltransferase
1,894,515	A → G	K9E	JYQ65_09770	Threonine/Serin exporter family protein

**Table 2:** Identification of insertion–deletion mutations in *L. fermentum* GR1009.

Location in PS150	Locus tag of PS150	Putative function	Length (bp)	Description
114,087 - 126,213	JYQ65_00485 to JYQ65_00545	Gene cluster for EPS biosynthesis	12,127	*eps1* gene cluster is absent in GR1009
208,756 - 208,764	JYQ65_00960	LPXTG cell wall anchor domain-containing protein	9	Pro273-Ile274-Met275 are absent in JYQ66_00895 of GR1009
401,025	JYQ65_01925	Acetyl-CoA carboxylase carboxyltransferase beta subunit	1,064	Insertion of an IS element in GR1009 (JYQ66_01860 to JYQ66_01870)
1,263,867 - 1,264,918	JYQ65_06440	IS30 family transposase	1,052	Deletion of an IS element in GR1009
1,362,783	-	-	1,471	Insertion of an IS element in GR1009 (JYQ66_06875)

### 3.5. A putative EPS biosynthetic gene cluster is disrupted in GR1009

**Figure 4:**
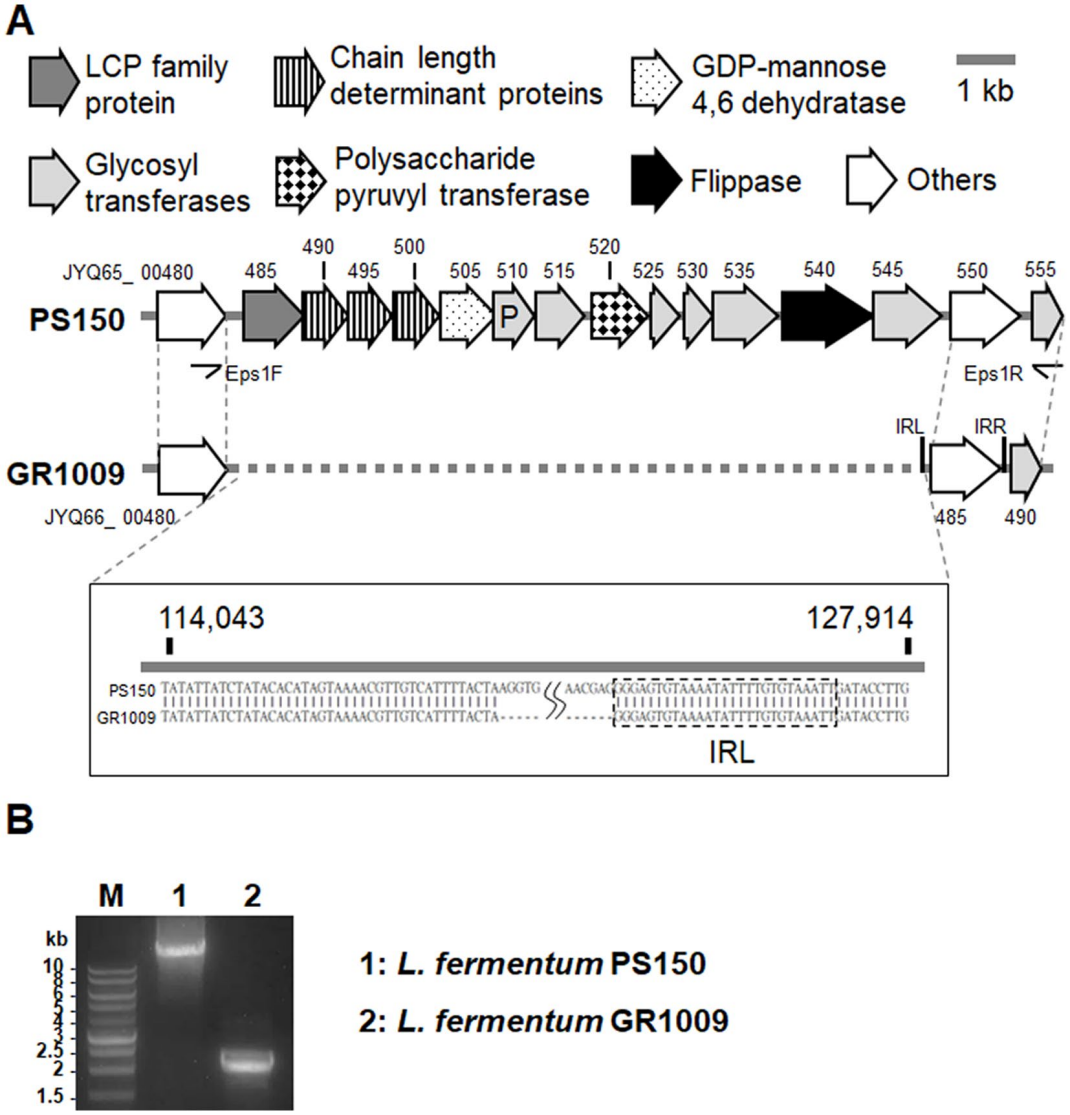
Comparison of the *eps1* gene region in *L. fermentum* PS150 and GR1009. **(A)** Schematic view of the EPS producing cluster in PS150 (112,500–128,000) and GR1009 (112,500–115,400). Detailed description of each locus tag is listed in Table 3. The deletion region of the EPS producing cluster in GR1009 was plotted with a dashed line. **(B)** PCR amplification of the *eps1* cluster region from PS150 or GR1009 with Eps1F/EPS1R primers. M, DNA molecular size marker.

As mentioned above, whole-genome sequencing revealed that GR1009 genome size is slightly smaller than PS150. Compared with PS150, a 13-kb deletion was detected in the GR1009 genome (Table 2). Functional annotation revealed that this deletion region in GR1009 is an EPS gene cluster (referred to as *eps1*) which contains 13 genes that fulfill the requirement for EPS biosynthesis, including a putative priming glycosyltransferase (GT), four GTs for sugar subunit synthesis, chain length determinant proteins, a polymerase for repeat unit synthesis, and a flippase for product exportation (Figure 4A; Table 3). We used polymerase chain reaction (PCR) and Sanger sequencing to reconfirm the deletion region and nucleotide sequence in PS150 and GR1009, respectively (Figure 4B). As shown in Figure 4A, a putative upstream inverted repeat (IRL), belonging to an IS256 transposable element (locus tag: JYQ66_00485), was detected adjacent to the deletion site. The results suggest that GR1009 might loss its esp1 cluster region during transposon jumping.

**Table 3:** Gene annotation of putative EPS producing cluster *eps1*.

Locus tag	BLAST^a^	Pfam^b^	RAST^c^
JYQ65_00480	IMP dehydrogenase	IMP dehydrogenase/GMP reductase domain	Inosine-5′-monophosphate dehydrogenase CBS domain
JYQ65_00485	LCP family protein	LytR_cpsA_psr cell envelope-related transcriptional attenuator domain	Cell envelope-associated transcriptional attenuator LytR-CpsA-Psr, subfamily F2
JYQ65_00490	Exopolysaccharide biosynthesis protein	Wzz Chain length determinant protein	Tyrosine-protein kinase transmembrane modulator EpsC
JYQ65_00495	CpsD/CapB family tyrosine-protein kinase	AAA domain	Tyrosine-protein kinase EpsD
JYQ65_00500	Exopolysaccharide biosynthesis protein	Not found	Manganese-dependent protein-tyrosine phosphatase
JYQ65_00505	GDP-mannose 4,6-dehydratase	GDP-mannose 4,6 dehydratase	UDP-glucose 4-epimerase
JYQ65_00510	Exopolysaccharide biosynthesis polyprenyl glycosylphosphotransferase	Bacterial sugar transferase	Sugar transferase
JYQ65_00515	Glycosyltransferase	Glycosyl transferase family 2	Putative glycosyltransferase
JYQ65_00520	Polysaccharide pyruvyl transferase family protein	Polysaccharide pyruvyl transferase	Hypothetical protein
JYQ65_00525	Glycosyltransferase	Glycosyl transferase family 2	Beta-1,3-glucosyltransferase
JYQ65_00530	Glycosyltransferase	Not found	Hypothetical protein
JYQ65_00535	Glycosyltransferase family 1 protein	Glycosyl transferases group 1	Glycosyltransferase
JYQ65_00540	Polysaccharide biosynthesis C-terminal domain-containing protein	Lipid II flippase MurJ	Hypothetical protein
JYQ65_00545	EpsG family protein	EpsG family	Hypothetical protein
JYQ65_00550	IS256 family transposase	Transposase, Mutator family	Mobile element protein

a https://blast.ncbi.nlm.nih.gov/Blast.cgi.

b http://pfam.xfam.org/null.

c https://rast.nmpdr.org/.

Given the discovery of *eps1* deletion in GR1009, we further assessed other potential EPS biosynthesis systems of PS150 by dbCAN2 prediction program.^1^ As the result, 11 carbohydrate-related gene clusters with various functions were identified in PS150, three of which featured multiple GTs, and possible exportation mechanisms were predicted as EPS biosynthetic gene clusters, including the *eps1*. The second putative EPS biosynthetic gene cluster (referred to as *eps2*) comprises four GTs and two transporters (Supplementary Table S2).

The third putative EPS biosynthetic gene cluster (referred to as *eps3*) comprises two glycosyltransferases for subunit synthesis, one polymerase for repeat unit polymerization, one chain length determinant protein, and a flippase for exportation (Supplementary Table S3). Sequence alignment revealed that both *eps2* and *eps3* of GR1009 were identical to that of PS150. Accordingly, loss of *eps1* was the only difference we found in the EPS biosynthetic related genes between both strains.

### 3.6. Measurement of EPS-producing levels in PS150 and GR1009

**Figure 5:**
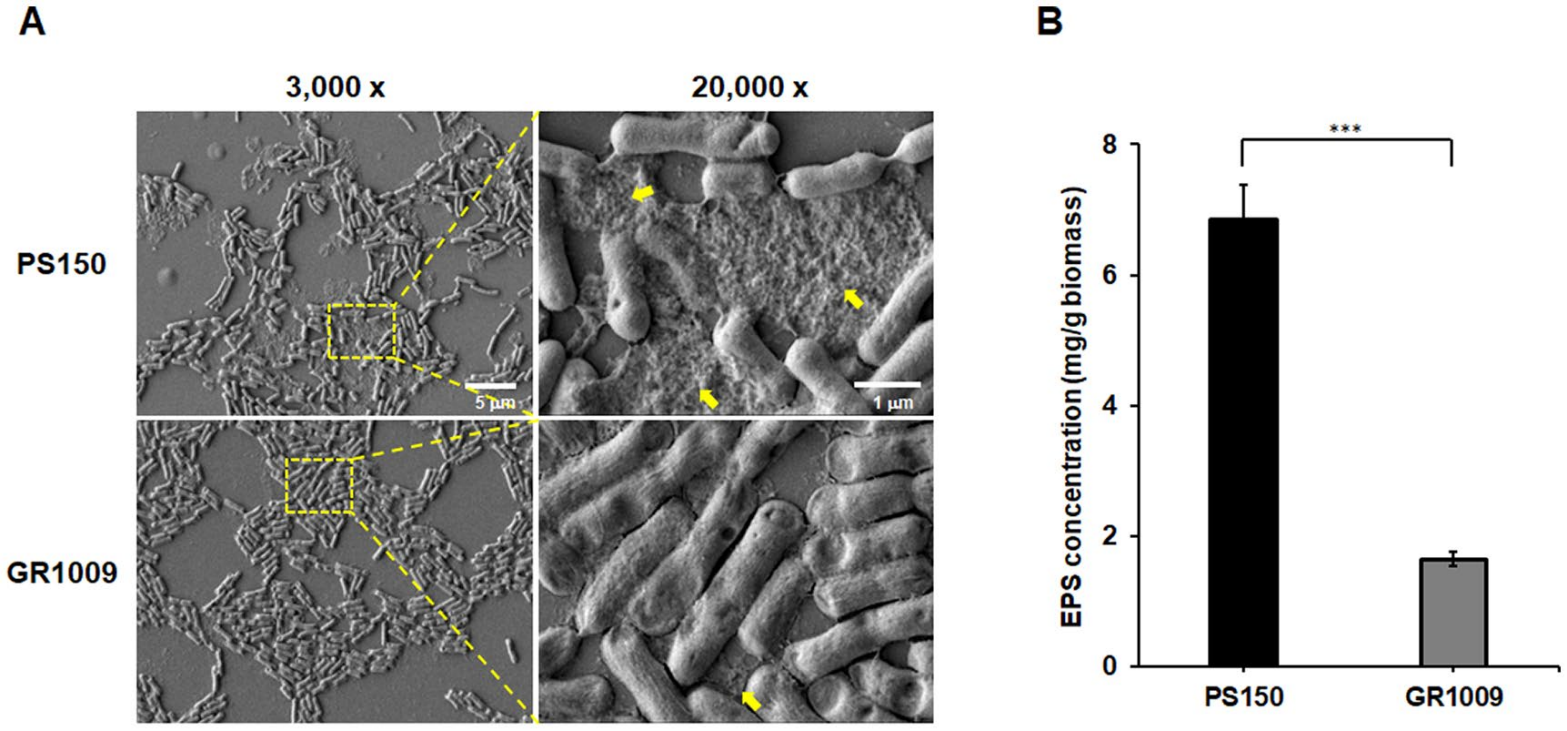
Comparative analysis of EPS levels produced by *L. fermentum* PS150 and GR1009. **(A)** The scanning electron microscopy (SEM) images of *L. fermentum* PS150 and GR1009 at different magnifications (3,000× and 20,000×). Yellow arrows indicate extracellular matrix material. **(B)** Sugar content in EPS matrix were quantified using carbohydrate assay with optical density (OD) 490 nm in 96-well plates. Data were expressed as mean ± standard error of the mean (SEM). ***p < 0.001 indicates statistical significance verified using an unpaired t-test.

To confirm the comparative approach results, PS150 and GR1009 were further tested for EPS production level. We used scanning electron microscopy (SEM) to investigate whether EPS was present in bacterial colony which obtained from the MRS agar plate. As shown in Figure 5A, we observed the presence of EPS-like matrix that heavily coated PS150 cells (yellow arrow), while GR1009 cells exhibited a relatively clean background, revealing potential differences in the EPS production level between PS150 and GR1009. To further qualify the EPS yield of PS150 and GR1009, we purified EPS-like matrix of PS150 and GR1009 from bacteria colonies on MRS agar plates and determined the sugar content by the phenol-sulfate method. As expected, PS150 contains a higher sugar content than GR1009. The EPS yield of GR1009 (1.65 ± 0.11 mg/g/biomass) was significantly lower than that of PS150 (6.84 ± 0.55 mg/g/biomass, *p* < 0.001; Figure 5B).

### 3.7. The C-EPS contains the sleep-improving effect in mice

**Figure 6:**
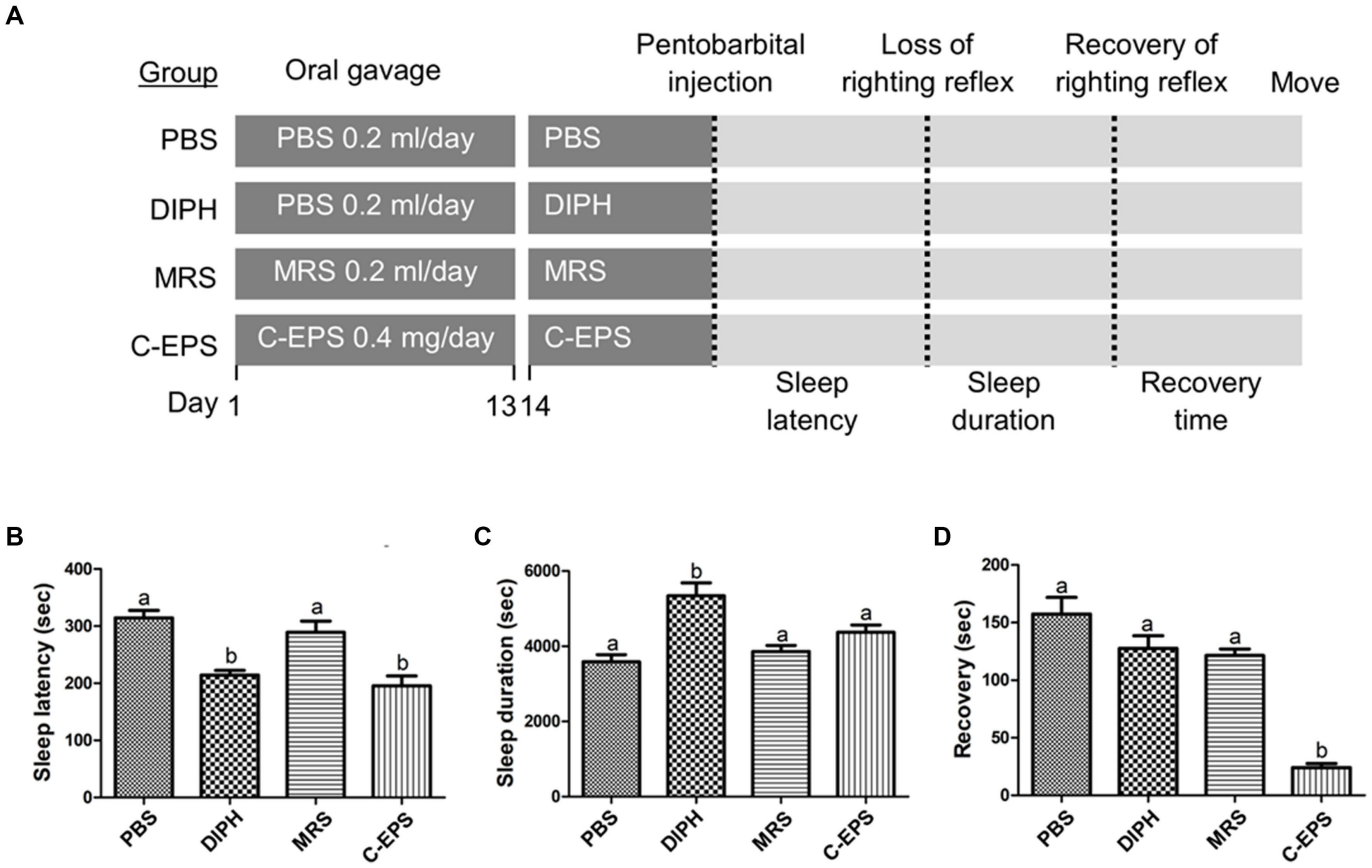
The hypnotic effect of exopolysaccharide-containing crude extracts from *L. fermentum* PS150 on pentobarbital-induced sleep in mice. **(A)** Schematic view of the experimental design. The unfermented Mann Rogosa Sharp (MRS) broth was used as the negative control. PBS, diphenhydramine (DIPH), MRS, and exopolysaccharide-containing crude extracts (C-EPS) were administered to the mice by oral gavage for 14 days, followed by the pentobarbital injection to evaluate sleep parameters. **(B–D)** Effects of C-EPS (*n* = 5 in each group) intervention on sleep latency, duration, and recovery. Different letters (a and b) above the columns indicate significant differences (*p* < 0.05) between the four groups. Any two treatments are assigned by the same letter at the top of the graphs, it indicates that there is no statistically significant difference. The comparisons were performed using one-way ANOVA with Tukey’s *post hoc* test (*p* < 0.05). The data are expressed as mean ± SEM.

As describe above, we demonstrated that GR1009 shown significantly lower EPS level than PS150. Based on the result, we further hypothesized that the EPS of PS150 is responsible for the reduced sleep recovery (Figure 6). To confirm the hypothesis, the hypnotic function of the EPS-containing crude extract (C-EPS) derived from PS150 was compared with PBS in a pentobarbital-induced sleep mouse model. Furthermore, we took unfermented MRS into comparison to ensure that the hypnotic function was not triggered by components in our culture medium. It showed significant reduction in sleep latency after treatment with the PS150-derived C-EPS [Figure 6B, *F*(3, 16) = 14.11, df = 3, *p* < 0.0001]. As live PS150 and HK-PS150, the C-EPS exhibited an increasing trend in terms of sleep duration [Figure 6C, *F*(3, 16) = 10.98, df = 3, *p* = 0.0004]. In addition, C-EPS treatment significantly reduced sleep recovery [Figure 6D, *F*(3, 16) = 35.29 df = 3, *p* < 0.0001]. The reduced sleep latency and recovery time revealed that the C-EPS was sufficient to reproduce the hypnotic effects of PS150. In contrast, the unfermented MRS did not affect any parameters in the present animal study. To sum up, our data demonstrated that GR1009 has lower EPS production level than PS150 due to *eps1* loss; most importantly, implied that EPS plays an important role in the hypnotic effect of PS150.

## 4. Discussion

The discovery of novel probiotic indications has grown in recent years (Kechagia et al., 2013; Kerry et al., 2018). Although most probiotics have been restricted to certain lactic acid bacterial species, the highly diverse nature of probiotic strains has hampered further research. Taking advantage of the modern omics approach, we demonstrated how a strain isolated from a common origin could be employed in a comparative study. The comparative multi-omics approach appeared to be extremely powerful in framing hypotheses with little information on probiotic strains. This strategy further emphasizes the importance of collecting and preserving bioresources when investigating novel probiotics.

In the present study, we proposed a possible connection between the hypnotic effect and the EPS synthesized by *eps1* of PS150. Featuring a flippase (locus_tag: JYQ65_00540) and a polymerase (locus_tag: JYQ65_00545), gene annotation revealed that eps1 is similar to the Wzx/Wzy-dependent EPS biosynthesis system (Zhou et al., 2019). The chain length determinant system of PS150 *eps1* encodes a Wzz family protein (locus_tag: JYQ65_00490), a tyrosine kinase (locus_tag: JYQ65_00495), and a phosphatase (locus_tag: JYQ65_00500), which is self-sustained for regulation. The three-component phosphorylation system has also been detected in other lactic acid bacteria, including *Lactobacillus johnsonii* (Horn et al., 2013), *Lactobacillus. Rhamnosus* (Lebeer et al., 2009), and other *L. fermentum strains* (Wei et al., 2019). The presence of four GTs in *eps1* further suggested a relatively complex repeat unit, possibly composed of heteropolysaccharides (Zhou et al., 2019). In contrast to the three-component system of *eps1*, *eps3* contains only one protein for chain length determination, which resembles the O-polysaccharide biosynthesis system in *Escherichia coli* (Woodward et al., 2010). Furthermore, only two GTs were identified in *eps3*, implying that the product of *eps3* is considerably simpler than *eps1*. On the one hand, the flippase (locus_tag: JYQ65_08645) and polymerase (locus_tag: JYQ65_08660) present in this cluster suggested that it is also Wzx/Wzy-dependent system (Supplementary Table S2). Conversely, *eps2* contains membrane transporter proteins instead of flippase and polymerase, suggesting that it belongs to the other biosynthesis system (Supplementary Table S1).

To identify possible genes related to hypnotic function, we hypothesized that the active component must meet three requirements: (i) it could be secreted to the extracellular; (ii) it must be heat-stable and remain soluble after heat-killing; (iii) it should be insoluble during ethanol precipitation and be resolved in water. Apart from the deletion of *eps1*, some SNPs and indels were also detected in the genomic analysis (Tables 1, 2). Considering SNPs, we ignored synonymous variants, intergenic variants, and genes with uncertain functional predictions, as there is no feasible method for subsequent biological validation. The remaining variants could affect the functions of the LPXTG cell wall anchor domain-containing protein, acetyl-CoA carboxylase biotin carboxylase subunit, insulinase, and threonine/serine exporter family proteins (Table 1). However, they were unlikely to be the active components themselves, as proteins are mostly heat-labile molecules that could be denatured during the heat-killing process. One of the indels resulted in the premature termination of the acetyl-CoA carboxylase carboxyl transferase beta subunit in GR1009 (Table 2). The truncation and point mutation of acetyl-CoA carboxylase subunits in GR1009 could lead to impaired malonyl-CoA production. Reduced malonyl-CoA availability may result in decreased malonyl-CoA-dependent molecules and fatty acid synthesis (Milke and Marienhagen, 2020). However, malonyl-CoA-dependent molecules and fatty acids are hydrophobic molecules, rendering them soluble during alcohol precipitation. Thus, we concluded that *eps1* deletion was the top candidate, regardless of other variants and indels.

EPS produced by lactic acid bacteria has long been recognized for its various bioactivities, including anti-oxidation, anti-lipogenic, anti-tumor, anti-hepatic steatosis, and anti-pathogen adhesion (Zivkovic et al., 2016; Trabelsi et al., 2017; Jiang and Yang, 2018; Wei et al., 2019; Zhang et al., 2019). Given our findings, we included the hypnotic effect as another potential application. Notably, it has been long proposed that the bacterial components may serve as somnogen, and the bacterial EPS was thus considered a potential hypnotic agent (Krueger et al., 1984; Nwodo et al., 2012). Although we linked the EPS of PS150 with the hypnotic effect, it remains unclear how EPS affects the host. EPS may carry out beneficial functions through four pathways: (i) it can be used as a prebiotic for commensal bacteria; (ii) it can compete with potential pathogens on the intestinal epithelium; (iii) it can regulate the expression of tight junction genes and improve barrier function; (iv) it can serve as an antigen to alter mucosal immunity of the gut (Nwodo et al., 2012). As the animal model employed in our study is not pathogenic and does not affect barrier function, EPS of PS150 is not likely to affect the host through these two pathways. Here, prebiotic and immunogenic pathways are potentially effective in the gut microbiota.

Previous studies showed that sleep fragmentation is associated with changes in the population of gut microbiota, multiple communication pathways are believed to exist within the gut-brain axis that can potentially regulate sleep, such as SCFAs, GABA, and serotonin (Szentirmai et al., 2019; Maki et al., 2020; Yu et al., 2020; Sen et al., 2021). In this study, we observed PS150 and HK-PS150 oral administration were significantly altered gut microbiome signatures in animal model (Supplementary Figure S1). Compared with PBS group, genus *Blautia* and *Dubosiella* increased in PS150 group or HK-PS150 group (Figure 3B); at the species level, *Lactobacillus fermentum* and *Clostridium* sp. *Culture* 54 levels both increased after the administration of PS150 or HK-PS150 (Figures 3C,D). Among of them, *Blautia*, as one of butyragenic genera, its level is correlated to circadian oscillation and has been reported being positively associated with sleep quality in young and healthy people (Grosicki et al., 2020; Koh et al., 2021; Ozato et al., 2022). Furthermore, *L. fermentum* have been demonstrated its butyrate-producing activity and sleep-improving effect *in vitro* and *in vivo* (Lin et al., 2019; Lacerda et al., 2022). *L. fermentum*-containing probiotic mixture can significantly improve the mood and sleep quality in clinical trial volunteers (Marotta et al., 2019; Dos Reis Lucena et al., 2021). Butyrate, a multi-functional molecule, is widely believed to improve brain function. In animal studies, butyrate was shown to accelerate brain-derived neurotrophic factor (BDNF) expression in the hippocampus via inhibition of histone deacetylase (Tu et al., 2017). Butyrate also shown anti-inflammatory effect in the bran by suppressing the TNF-α synthesis (Noble et al., 2017). Butyrate supplementation significantly improved behavioral abnormalities and modulated microglia homeostasis in mice (Duan et al., 2021). Based on these findings, the hypnotic effect of PS150 and HK-PS150 in terms of sleep may be partially explained by microbiota modulation.

Collectively, the present study provides morphological and genetic evidence linking the EPS of PS150 with its hypnotic effect. Subsequently, using an animal study accompanied by chemical characterization, we suggested that C-EPS, a heteropolysaccharide-containing extract, might exhibit hypnotic effects. Our study suggests the potential use of purified EPS derived from PS150 as a hypnotic drug. However, further studies on EPS, including its chemical structure and biological mechanism, are warranted in the future.

## Data availability statement

The datasets presented in this study can be found in online repositories. The names of the repository/repositories and accession number(s) can be found at: NCBI Bioproject (PRJNA702613).

## Ethics statement

The animal study was reviewed and approved by Institutional Animal Care and Use Committee of the National Yang Ming Chiao Tung University.

## Author contributions

C-LH and H-FC: conceptualization. C-LH, H-FC, and C-CW: study design. C-LH: animal model. H-FC and F-SD: EPS purification. P-JW: SEM images. C-HC and M-KL: EPS analysis. H-FC, S-PC, and Y-TC: bioinformatic analysis. C-LH, H-FC, and Y-TC: original draft. C-CW: editing. Y-CT: supervising. All authors contributed to the article and approved the submitted version.

## Funding

The authors declare that this study received funding from Bened Biomedical Co., Ltd. The funder was not involved in the study design, collection, analysis, interpretation of data, the writing of this article, or the decision to submit it for publication.

## Conflict of interest

C-LH, P-JW, F-SD, and C-CW are employed by Bened Biomedical Co., Ltd. Y-CT is a stockholder and a consultant of Bened Biomedical Co., Ltd.

The remaining authors declare that the research was conducted in the absence of any commercial or financial relationships that could be construed as a potential conflict of interest.

## Publisher’s note

All claims expressed in this article are solely those of the authors and do not necessarily represent those of their affiliated organizations, or those of the publisher, the editors and the reviewers. Any product that may be evaluated in this article, or claim that may be made by its manufacturer, is not guaranteed or endorsed by the publisher.

## Footnotes

^1^ https://bcb.unl.edu/dbCAN2/
