# Supplementary material for: Exopolysaccharide is the potential effector of Lactobacillus fermentum PS150, a hypnotic psychobiotic strain
Source: Front Microbiol. 2023 Jul 3;14:1209067. doi: 10.3389/fmicb.2023.1209067 (PMC10352126; doi:10.3389/fmicb.2023.1209067)
Supplement: Supplementary file 1 [file Data_Sheet_1.docx]

Supplementary Material

Exopolysaccharide is the Potential Effector of *Lactobacillus fermentum* PS150, a Hypnotic Psychobiotic Strain

Chin-Lin Huang^1,2^, Hsu-Feng Chu^1^, Chien-Chen Wu^2^, Fu-Sheng Deng^2^, Pei-Jun Wen^2^, Shao-Ping Chien^3^, Chi-Hsein Chao^4^, Ying-Tsong Chen^3^, Mei-Kuang Lu^4^, and Ying-Chieh Tsai^5*^

*** Correspondence:** Ying-Chieh Tsai: tsaiyc@ym.edu.tw

# Supplementary Tables

| Strains | Genome size | Total coverage | GC content | No. of CDSs | GenBank number |
| --- | --- | --- | --- | --- | --- |
| PS150 | 2,237,764 | 115.3 | 51% | 2,204 | CP071000.1 |
| GR1009 | 2,227,144 | 101.5 | 51% | 2,195 | CP070999.1 |

**Table S1.** Amount of genome sequence data for *L. fermentum* PS150 and GR1009

| Locus tag | | | Function | | Carbohydrate-active enzyme* |
| --- | --- | --- | --- | --- | --- |
| PS150 | GR1009 | |  |  |  |
| JYQ65_08525 | JYQ66_8470 | IS200/IS605 family transposase | | - | |
| JYQ65_08530 | JYQ66_8475 | Hypothetical protein | | - | |
| JYQ65_08535 | JYQ66_8480 | ABC transporter ATP-binding region | | - | |
| JYQ65_08540 | JYQ66_8485 | GntR family transcriptional regulator | | - | |
| JYQ65_08545 | JYQ66_8490 | dTDP-glucose 4,6-dehydratase | | - | |
| JYQ65_08550 | JYQ66_8495 | Hypothetical protein | | - | |
| JYQ65_08555 | JYQ66_8500 | Glycosyltransferase family 2 protein | | Glycosyltransferase family 2 | |
| JYQ65_08560 | JYQ66_8505 | Sugar transferase | | - | |
| JYQ65_08565 | JYQ66_8510 | Peptide chain release factor 3 | | - | |
| JYQ65_08570 | JYQ66_8515 | Transposase | | - | |
| JYQ65_08575 | JYQ66_8520 | Glycosyltransferase family 2 protein | | Glycosyltransferase family 2 | |
| JYQ65_08580 | JYQ66_8525 | AI-2E family transporter | | - | |
| JYQ65_08585 | JYQ66_8530 | Glycosyltransferase family 2 protein | | Glycosyltransferase family 2 | |
| JYQ65_08590 | JYQ66_8535 | Hypothetical protein | | - | |
| JYQ65_08595 | JYQ66_8540 | C40 family peptidase | | Glycoside hydrolase family 73  Carbohydrate-binding module family 50 | |

*Gene annotations based on Carbohydrate-Active enzymes (CAZy) database.

**Table S2.** Gene annotation of putative EPS biosynthesis gene cluster *eps2*

| Locus tag | | Function | Carbohydrate-active enzyme* |
| --- | --- | --- | --- |
| PS150 | GR1009 |  |  |
| JYQ65_08620 | JYQ66_8565 | IS256 family transposase | - |
| JYQ65_08625 | JYQ66_8560 | Hypothetical protein | - |
| JYQ65_08630 | JYQ66_8555 | IS30 family transposase | - |
| JYQ65_08635 | JYQ66_8550 | Glucosaminidase domain-containing protein | Glycoside hydrolase family 73  Carbohydrate-binding module family 50 |
| JYQ65_08640 | JYQ66_8545 | Acyltransferase | - |
| JYQ65_08645 | JYQ66_8540 | Flippase | - |
| JYQ65_08650 | JYQ66_8535 | UDP-galactopyranose mutase | - |
| JYQ65_08655 | JYQ66_8530 | Lipopolysaccharide biosynthesis protein | - |
| JYQ65_08660 | JYQ66_8525 | Polymerase | - |
| JYQ65_08665 | JYQ66_8520 | DUF4422 domain-containing protein | Glycosyltransferase family 111 |
| JYQ65_08670 | JYQ66_8515 | Sugar transferase | - |
| JYQ65_08675 | JYQ66_8510 | Recombination regulator RecX | - |
| JYQ65_08680 | JYQ66_8505 | Hypothetical protein | - |
| JYQ65_08685 | JYQ66_8500 | DUF2922 domain-containing protein | - |

**Table S3.** Gene annotation of putative EPS biosynthesis gene cluster *eps3*

*Gene annotations based on Carbohydrate-Active enzymes (CAZy) database.

##
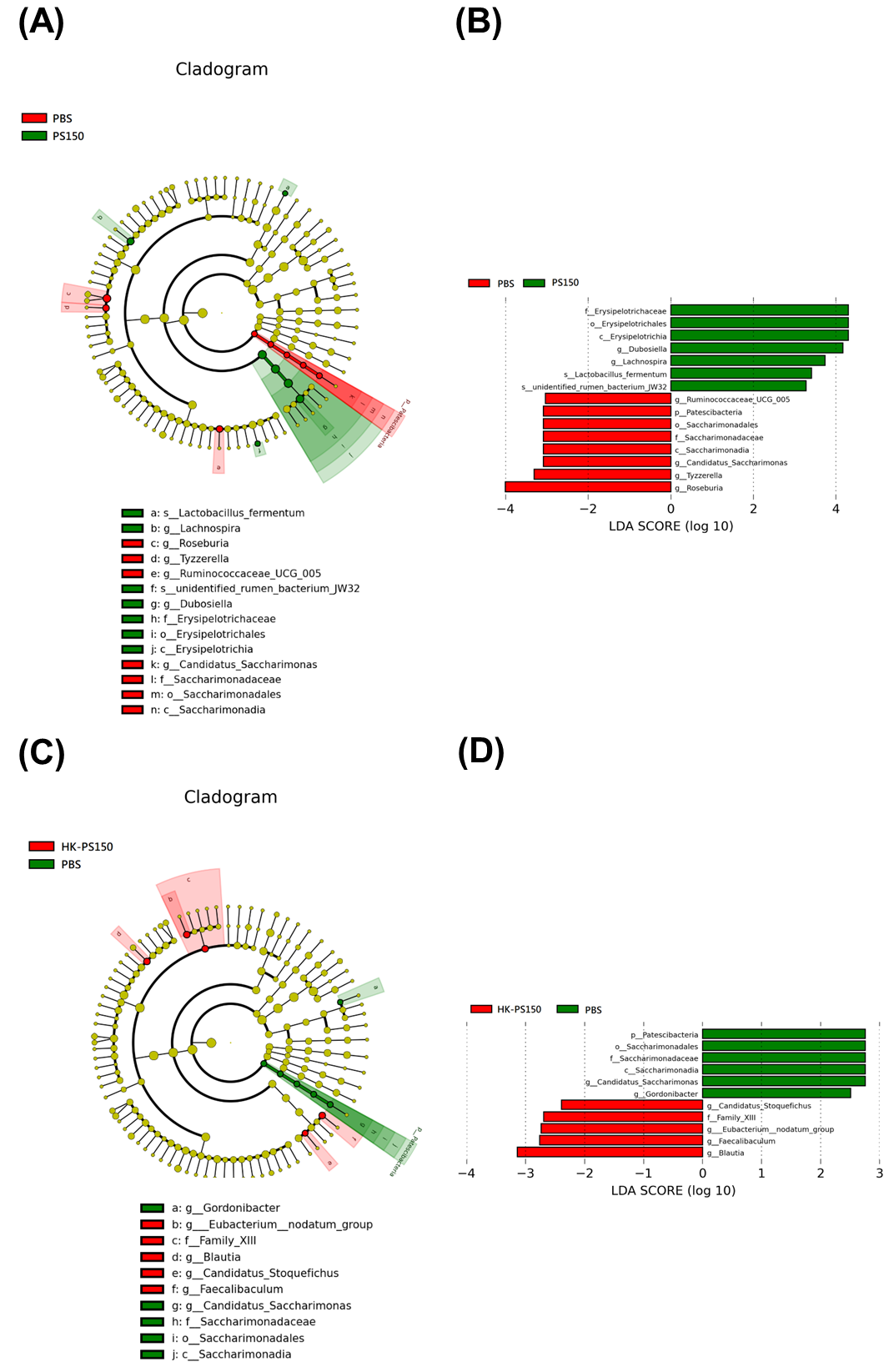
Supplementary Figure

**Figure S1.** Comparison of the gut microbiome composition in PBS, PS150 and HK-PS150. **(A)** Cladogram plotted in red for the enriched taxonomy in PBS mice and in green for the enriched taxonomy in PS150 mice. **(B)** Linear discriminant analysis (LDA) effect size showing significant differences between PBS and PS150 groups. **(C)** Cladogram plotted in green for the enriched taxonomy in PBS mice and in red for the enriched taxonomy in HK-PS150 mice. **(D)** LDA effect size showing significant differences between PBS and HK-PS150 groups. The length of the bar represents the LDA score (taxa with LDA score > 2).
